# The Impact of Tenapanor on Serum Phosphate in Adult Dialysis Patients: A Narrative Review

**DOI:** 10.1016/j.xkme.2025.101237

**Published:** 2026-01-03

**Authors:** Malki Waldman, Stephani Johnson, Melanie Newkirk, Corey Hawes, Laura Byham-Gray

**Affiliations:** Department of Clinical and Preventive Nutrition Sciences, School of Health Professions, Rutgers University, Newark, NJ

**Keywords:** Tenapanor, sodium hydrogen exchange, dialysis, end-stage kidney disease, hyperphosphatemia

## Abstract

Managing hyperphosphatemia remains a challenging feat in the dialysis population. In 2023, tenapanor, a sodium hydrogen exchanger inhibitor, was approved in the United States for treating hyperphosphatemia in adults with chronic kidney disease receiving dialysis. This narrative review evaluated the recent literature on tenapanor, its effects on serum phosphate, and the achievement of target serum phosphate reference ranges in individuals undergoing peritoneal and hemodialysis. In addition to efficacy, this review examined the prevalence of drug-induced diarrhea. Twelve articles met the inclusion criteria. Results indicate clinical and statistical differences in the mean change in serum phosphate and a greater tendency to achieve serum phosphate target reference ranges among individuals receiving dialysis who take tenapanor compared with those who do not. These findings support using tenapanor independently or in conjunction with phosphate binders to treat hyperphosphatemia. However, drug-induced diarrhea may lead to discontinuation of tenapanor therapy. Future research should focus on nutrition counseling to mitigate drug-induced diarrhea and extend tenapanor tolerance in individuals receiving dialysis.

## Introduction

The prevalences of hyperphosphatemia in the US Chronic Kidney Disease (CKD) stage 5 hemodialysis and peritoneal dialysis populations are 42% and 46%, respectively.^1^ Hyperphosphatemia poses a significant concern because of its association with an increased risk of vascular calcification, morbidity, and mortality.^2^ The CKD stage 5 population is prone to hyperphosphatemia because the kidneys maintain phosphate homeostasis through vitamin D, intact parathyroid hormone, fibroblast growth factor 23, the brain, and the gut.^3^ When kidney function declines in CKD stage 5, the dysregulation between these components results in phosphate retention and hyperphosphatemia.^4^ The current practice in dialysis facilities aimed to reduce hyperphosphatemia through dietary modification, dialysis, and medication.^5^

The conventional American diet consists of ∼1,500 mg of phosphate a day.^6^ Approximately 800 mg of phosphate is absorbed in the gastrointestinal tract daily,^7^ but it varies owing to dietary phosphate source and type.^2^ Hemodialysis removes roughly 2.4-3.6 g of phosphate weekly,^4^ whereas peritoneal dialysis only removes 2.1-2.5 g weekly.^7^ Reliance on dietary modification to control hyperphosphatemia is challenging because of the rampant phosphate additives with significantly higher bioavailability than natural phosphate sources.^8^ Despite the requirement for dialysis units to have a dietitian to provide evidence-based nutrition counseling,^9^ achieving serum phosphate levels within the reference range (2.5-4.5 mg/dL)^10^ remains elusive for many dialysis patients.^11^^,^^12^

In addition to a low-phosphate diet, a routine dialysis prescription often includes phosphate-lowering medications that prevent gastrointestinal absorption of ∼300 mg of phosphate daily.^13^ Most phosphate binders contain a cation that binds to the anionic phosphate in the stomach or proximal small intestine, creating a nonabsorbable compound excreted via the stool.^14^ An exception to that is sevelamer, which is an anion exchange resin.^14^ Suboptimal effectiveness,^11^ a high pill burden,^14^ gastrointestinal side effects,^14^ and calcium and metallic loads^15^ make phosphate binders less than ideal options. Recent drug trials exploring the safety and efficacy of tenapanor in managing hyperphosphatemia in dialysis patients may provide an alternative to phosphate binders.^16^, ^17^, ^18^, ^19^, ^20^, ^21^, ^22^, ^23^, ^24^, ^25^, ^26^, ^27^

Tenapanor was developed in 2008 by Ardelyx.^28^ Although research evaluated the impact of tenapanor on irritable bowel syndrome (IBS) with constipation,^29^ further investigation explored its effects on hyperphosphatemia in adults with CKD.^18^ The Food and Drug Administration approved tenapanor for treating IBS with constipation in 2019^30^^,^^31^ and hyperphosphatemia in 2023.^28^ Tenapanor inhibits the sodium hydrogen exchanger isoform 3, thereby increasing intracellular hydrogen ions in the intestinal epithelial cells.^32^ This inhibition leads to a conformational change in the tight junction proteins, preventing paracellular phosphate absorption.^33^ With its novel approach, tenapanor may offer ideal serum phosphate control without the iatrogenic effects of phosphate binders. However, the excess sodium ions in the intraluminal space of the intestines promote loose stools.^32^ Given the widespread prevalence of gastrointestinal issues in the CKD population,^11^^,^^34^ it is imperative to evaluate the merits of using this medication. Given the limited data, this narrative review explores preliminary evidence of the efficacy of tenapanor in managing hyperphosphatemia and achieving target serum phosphate levels in adults receiving dialysis, as well as the reported occurrence of drug-induced diarrhea.

## Literature Search

In February 2024, a literature search encompassed 4 databases (PubMed, CINAHL, Embase, and Scopus) and was limited to publications in the past 5 years written in English. Keywords included “tenapanor,” “dialysis,” and “phosphate.” Inclusion criteria were clinical or randomized controlled trials and studies with more than 10 individuals in each group. Studies were excluded if the population was aged <18 years, included pregnant women, or had a study group of <10 individuals. Additional exclusion criteria were studies published in a non-English language, case reports, or observational in design. Studies with the same authorship were eligible for inclusion if they provided original content. Although a study dropout rate of >20% is typically appropriate, in the context of drug trials, the dropout rate was not a criterion for exclusion but rather a study limitation.

**Figure 1:**
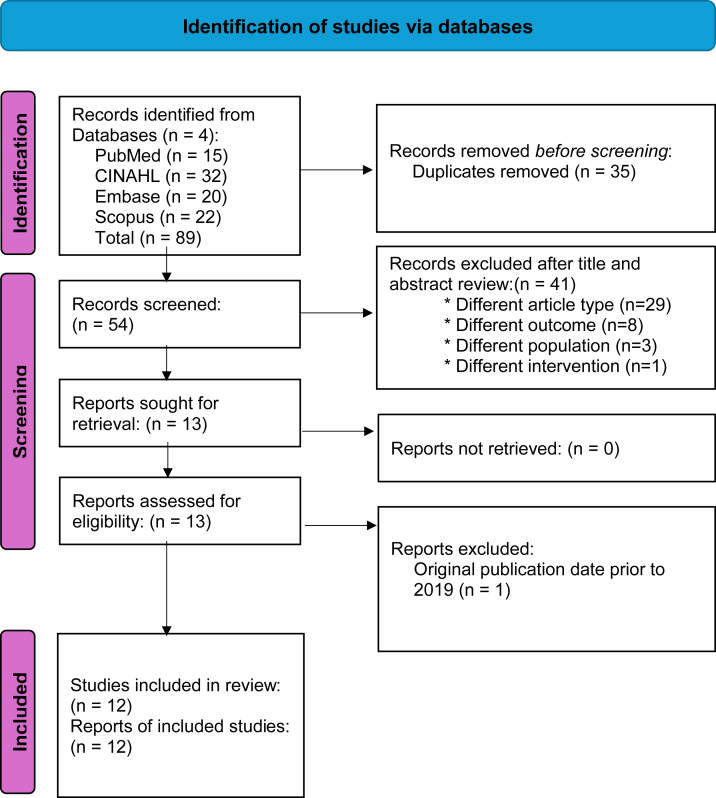
Preferred Reporting Items for Systematic Reviews and Meta-Analyses diagram (Page et al^35^).

The search identified 89 articles; 76 were excluded based on title and abstract screening because of duplication, study designs, irrelevant outcomes, animal studies, or different interventions. The remaining 13 articles were retrieved and reviewed in full text. One article did not meet the inclusion criteria because of the original publication date. All other retrieved studies met the eligibility requirements. Refer to the Preferred Reporting Items for Systematic Reviews and Meta-Analysis flow diagram in Fig 1 for the search methodology.^35^

## Results

Of the studies included in this review, 6 occurred in Japan,^16^^,^^20^, ^21^, ^22^, ^23^^,^^25^ 1 in China,^19^ and 5 in the United States.^17^^,^^18^^,^^24^^,^^26^^,^^27^ All Japanese studies had a minimum age of 20 years,^16^^,^^20^, ^21^, ^22^, ^23^^,^^25^ whereas the American^17^^,^^18^^,^^24^^,^^26^^,^^27^ and Chinese^19^ studies had a minimum age of 18 years. The minimum dialysis vintage was 3 months for those receiving hemodialysis^16^, ^17^, ^18^, ^19^, ^20^, ^21^^,^^23^, ^24^, ^25^, ^26^, ^27^ and 3^22^ to 6 months^17^^,^^24^^,^^26^^,^^27^ for peritoneal dialysis. All studies had a qualifying serum phosphate range on screening and subsequent study periods to maintain participation.^16^, ^17^, ^18^, ^19^, ^20^, ^21^, ^22^, ^23^, ^24^, ^25^, ^26^, ^27^

On screening, the required serum phosphate ranges were 6.1-10 mg/dL,^20^^,^^23^^,^^25^ 3.5-7.0 mg/dL,^16^^,^^21^^,^^22^ 4.0-7.0 mg/dL,^18^^,^^19^ 4.0-8.0 mg/dL,^17^^,^^26^ and 5.5-10.0 mg/dL.^24^^,^^27^ One study had a broader eligible serum phosphate range of 4.5-10.0 mg/dL for participants not on phosphate binders.^27^ In addition, some studies required an increase in serum phosphate after a washout of at least 1.0 mg/dL^20^ or 1.5 mg/dL.^17^, ^18^, ^19^^,^^26^ Finally, eligibility criteria for most studies included a serum phosphate range after a washout of 3.5-7.0 mg/dL,^16^ 6.0-10 mg/dL,^17^, ^18^, ^19^^,^^26^ 6.1-10.0 mg/dL,^22^^,^^23^^,^^25^ or 5.5-10.0 mg/dL.^24^

Investigators required pre-existing phosphate binder prescriptions to be stable or regulated by study parameters.^16^, ^17^, ^18^, ^19^, ^20^, ^21^, ^22^, ^23^, ^24^, ^25^, ^26^, ^27^ Similarly, most studies restricted changes in established vitamin D and calcimimetic prescriptions for 2^16^^,^^23^^,^^25^ to 4^18^, ^19^, ^20^, ^21^, ^22^^,^^24^ weeks before screening. Most trials required Kt/V urea ≥ 1.2 as a condition for enrollment.^17^^,^^19^, ^20^, ^21^^,^^23^, ^24^, ^25^, ^26^, ^27^ The main exclusion criteria were pre-existing diarrhea,^16^^,^^18^, ^19^, ^20^, ^21^, ^22^, ^23^^,^^25^ IBS, IBS with diarrhea,^16^^,^^17^^,^^19^, ^20^, ^21^, ^22^, ^23^, ^24^, ^25^, ^26^, ^27^ or elevated parathyroid levels, either >600 pg/mL^16^^,^^20^^,^^21^^,^^23^^,^^25^ or >1,200 pg/mL.^17^, ^18^, ^19^^,^^24^^,^^26^^,^^27^

Recruitment periods for the trials occurred between 2016 and 2023.^16^, ^17^, ^18^, ^19^, ^20^, ^21^, ^22^, ^23^, ^24^, ^25^, ^26^, ^27^ Participants who received at least 1 drug dose and had a serum phosphate measurement comprising the intention-to-treat data sets. The total number of participants in the intention-to-treat data sets ranged from 43^16^-564.^17^ The Japanese studies had mean ages between 59 and 65 years,^16^^,^^20^, ^21^, ^22^, ^23^^,^^25^ and the trial conducted in China had a younger mean age of 54 years.^19^ Comparatively, the studies in the United States had mean ages in the 50s.^17^^,^^18^^,^^24^^,^^26^^,^^27^ Male patients made up the majority of participants.^16^, ^17^, ^18^, ^19^, ^20^, ^21^, ^22^, ^23^, ^24^, ^25^, ^26^, ^27^ Table 1 displays the literature review.^16^, ^17^, ^18^, ^19^, ^20^, ^21^, ^22^, ^23^, ^24^, ^25^, ^26^^,^^36^

**Table 1:** Safety and Efficacy of Tenapanor in Dialysis Patients

Author, Year, Design, Country, Funding Source, Quality Grade	Study Purpose	Study Population	Intervention	Outcome Data	Conclusions/Results	Limitations of Findings
Akizawa et al,^16^ open-label, single-arm study, multicenter, Japan Funded by Kyowa Kirin Co Ltd Conducted between December 2018 and November 2019 Quality grade^a^: +	To study the effectiveness of TNP on reducing PB pill burden and managing Sp	92 Consented, 25 excluded, 67 enrolled, 43 completed the study $\bar{X\;}$ age (SD): 62.7 y (10.2), 63% male Inclusion criteria: aged 20-79 y, HD 3 times a week, dialysis vintage ≥ 12 wk, unchanged doses of vitamin D and calcimimetic, Sp ≥ 3.5 mg/dL and ≤ 7.0 mg/dL at screening and during observation period Exclusion criteria: iPTH > 600 pg/mL, history of IBD or IBS with diarrhea, presence of diarrhea Discontinuation rate: 35.8%	TNP 30 mg twice a day + PB for 26 wk Final TNP dose after titration: ∼50% remained on 30 mg and the rest titrated to 20 mg, 10 mg, or 5 mg Data assessed: patients (%) with ≥30% decrease in total PB/TNP tablets wk 24-26 vs BL patients (%) with ≥30% decrease in total PB/TNP tablets at each time point vs BL Δ in Sp at each time point compared with BL Safety (AE)	At wk 24-26: 72% had ≥30% decrease in total PB and TNP tablets as compared with BL (95% CI, 59.3-82.0; *P* < 0.001) 52% had ≥50% decrease in total PB and TNP tablets as compared with BL (95% CI, 39.7-64.6; *P* < 0.001) 28% completely switched from PB to TNP tablets (95% CI, 18.0-40.7; *P* < 0.001) At wk 26, $\bar{X\;}$ Δ in Sp as compared with BL: -0.40 mg/dL AE diarrhea: 76%	Adding TNP to PB decreased pill burden and maintained Sp control	Open label, no placebo used for comparison Multiple PB–variety of doses and number of pills Study location limits generalizability to United States owing to cultural differences in diet
Shigematsu et al,^25^ randomized, double-blind, placebo-controlled trial, phase II, multicenter, Japan Funded by Kyowa Kirin Co Ltd Recruited between April 2019 and December 2019 Quality grade: +	To explore the efficacy and safety of TNP as dual-mechanism therapy in Japanese HD patients who are on PB therapy with hyperphosphatemia	133 Consented, 68 pre-enrolled, 47 randomized, 38 completed the study PCO + PB (n = 24) $\bar{X}$ age (SD): 59.4 y (9.2), 62.5% male TNP + PB (n = 23) $\bar{X}$ age (SD): 62.1 y (8.5), 82.6% male Inclusion criteria: aged ≥20 y and <80 y, HD 3 times a week, dialysis vintage ≥ 12 wk, PB 3 times a day, dose unchanged for the preceding 2 wk, Sp ≥ 6.1 mg/dL and < 10 mg/dL Exclusion criteria: PTH > 600 pg/mL, history of IBD or IBS with diarrhea, or presence of diarrhea Discontinuation rate: 19.1% PCO + PB: (n = 2) TNP + PB: (n = 7)	TNP 30 mg + PB or PCO + PB twice a day for 6 wk Final TNP twice a day dose after titration: 30 mg (n = 8) 20 mg (n = 4) 10 mg (n = 1) 5 mg (n = 3) Data assessed: Δ in Sp at wk 6 compared with BL Achievement of Sp ≤ 6.0 or ≤ 5.5 mg/dL Safety (AE)	TNP + PB showed greater improvement in reducing Sp than PB alone ($\bar{X}$ Δ in PCO + PB 0.08 mg/dL vs -1.99 mg/dL in TNP + PB, with a between-group difference of -2.07 (95% CI, -2.89 to -1.26; *P* < 0.001) TNP + PB had a greater achievement rate of Sp ≤ 6.0 mg/dL than PCO + PB at wk 6 (87% vs 37.5%) TNP + PB had a greater achievement rate of Sp ≤ 5.5 mg/dL than PCO + PB at wk 6 (73.9% vs 25%) AE diarrhea: TNP + PB: 65.2% PCO + PB: 8.3%	TNP is an effective add-on therapy in individuals with refractory hyperphosphatemia	Majority of patients in both PCO + PB (66.7%) and TNP + PB (82.6%) used calcium-based PBs, results may be limited to other PBs Small sample size Short treatment duration of 6 wk Study location limits generalizability to United States owing to cultural differences in diet
Inaba et al,^20^ phase 2, randomized trial, double-blind, placebo-controlled, parallel-group, dose-finding study, multicenter, Japan Funded by Kyowa Kirin Co Conducted between April 2019 and December 2019 Quality grade: +	To evaluate safety and efficacy of TNP and determine the clinically recommended dose by comparing change in Sp and safety outcomes at the end of study with BL	384 Consented, 283 pre-enrolled, 207 randomized, 141 completed second washout period PCO $\bar{X\;}$ age (SD): 63.9 y (10.5), 68.3% male TNP 5 mg $\bar{X\;}$ age (SD): 63.6 y (10.4), 61.9% male TNP 10 mg $\bar{X\;}$ age (SD): 65.4 y (8.8), 68.3% male TNP 30 mg $\bar{X\;}$ age (SD): 63.1 y (9.2), 71.4% male TNP 30-mg titration $\bar{X\;}$ age (SD): 61.7 y (10.0), 65.9% male Inclusion criteria: aged 20-80 y, HD 3 times a week, dialysis vintage ≥ 12 wk, unchanged dialysis Rx in the past 2 wk, unchanged PB (at least 3 times a day) for the past 4 wk, Kt/V ≥ 1.2, unchanged vitamin D and calcimimetics in the past 4 wk, Sp ≥ 6.1 mg/dL and < 10 mg/dL and had to increase ≥ 1.0 mg/dL during the first washout period Exclusion criteria: PTH > 600 pg/mL, IBD or IBS with diarrhea, diarrhea, GI surgery, additional exclusion criteria listed in supplementary materials Discontinuation rate in treatment period: 22.7% PCO: (n = 12) TNP 5 mg: (n = 5) TNP 10 mg: (n = 10) TNP 30 mg: (n = 13) TNP 30-mg titration: (n = 7)	TNP or PCO twice a day for 6 wk PCO (n = 41) TNP 5 mg twice a day (n = 42) TNP 10 mg twice a day (n = 41) TNP 30 mg twice a day (n = 42) TNP 30-mg twice a day titration (n = 41) Final TNP dose at wk 6 in TNP 30-mg twice a day titration grp: TNP 30 mg: 71.4% TNP 20 mg: 11.4% TNP 10 mg: 14.3% TNP 5 mg: 2.9% Data assessed: Δ in Sp at wk 6 compared with BL Δ in Sp at each time point compared with BL Achievement of Sp ≤ 6.0 mg/dL or ≤ 5.5 mg/dL Safety (AE)	$\bar{X\;}$ Δ in Sp at wk 6 compared with BL (mg/dL): PCO: 0.6 TNP 5 mg: -0.9 TNP 10 mg: -1.4 TNP 30 mg: -1.9 TNP 30-mg titration: -2.0 Sp significantly declined at wk 6 compared with BL in all grps vs PCO (*P* < 0.001) TNP 5 mg: -1.6 TNP 10 mg: -2.0 TNP 30 mg: -2.6 TNP 30-mg titration: -2.6 TNP had a greater achievement rate of sP ≤ 6.0 mg/dL than PCO at wk 6: PCO: 12.2% TNP 5 mg: 40.5% TNP 10 mg: 43.9% TNP 30 mg: 66.7% TNP 30-mg titration: 70.7% AE diarrhea: PCO: 9.8% TNP 5 mg: 50% TNP 10 mg: 65.9% TNP 30 mg: 76.2% TNP 30-mg titration: 65.9%	Tenapanor at a dose as low as 5 mg twice a day produced significant reductions in Sp and a greater frequency of achieving target reference ranges as compared with placebo	Short treatment duration of 6 wk Study location limits generalizability to United States owing to cultural differences in diet
Nitta et al,^23^ randomized, double-blind, placebo-controlled, parallel-group study, phase 3 trial, multicenter, Japan Funded by Kyowa Kirin Co Ltd Conducted between April 2021 and September 2021 Quality grade: +	To evaluate safety and efficacy of TNP with uptitration when added to PB in Japanese HD patients with hyperphosphatemia	210 Screened, 41 excluded, 169 enrolled, 147 completed the study PCO + PB (n = 85) TNP + PB (n = 84) mITT: TNP + PB $\bar{X\;}$ age (SD): 61.5 y (11.19), 63% male PCO + PB $\bar{X\;}$ age (SD): 60.6 y (11.03), 66% male Inclusion criteria: aged ≥20 y, HD 3 times a week, dialysis vintage ≥ 12 wk, PB dose unchanged in past 2 wk, Sp ≥ 6.1 mg/dL and < 10 mg/dL at screening and after 1-2 wk after run-in period Exclusion criteria: PD within past 12 wk, iPTH > 600 pg/mL, IBD or IBS with diarrhea, GI surgery within past 3 mo, severe heart disease or hepatic dysfunction, uncontrolled HTN or DM, upcoming kidney transplant, change in modality, life expectancy ≤ 12 mo Discontinuation rate: 13% TNP + PB: 13.1% PCO + PB: 12.9%	TNP 5 mg + PB or PCO + PB twice a day for 8 wk TNP dose after titration at wk 7: 5 mg: 39.2% 10 mg: 20.3% 20 mg: 16.2% 30 mg: 23.0% Data assessed: $\bar{X\;}$ Δ in Sp from BL compared with wk 8 and each time point Achievement of Sp between 3.5 and 6.0 mg/dL Safety (AE)	$\bar{X\;}$LS Δ from BL to wk 8 in Sp was -0.24 in PCO + PB and -2.00 mg/dL in TNP + PB Statistically significant difference between grps (-1.76; 95% CI, -2.16 to -1.37 mg/dL, *P* < 0.0001) TNP + PB had a greater achievement rate of Sp between 3.5 mg/dL and 6.0 mg/dL than PCO + PB at endpoint: TNP + PB: 70.4% PCO + PB: 28.9% AE diarrhea: TNP + PB: 63.1% PCO + PB: 14.1%	TNP is a treatment option for uncontrolled hyperphosphatemia in Japanese HD patients	Short treatment duration of 8 wk Majority of patients used multiple PB limiting insight on impact of individual PB Majority of study patients used hemodiafiltration dialysis Study location limits generalizability to United States owing to cultural differences in diet
Koiwa et al,^21^ open-label, single-arm, phase 3 trial, multicenter, Japan Funded by Kyowa Kirin Co Ltd Conducted between March 2021 and June 2022 Quality grade: +	To evaluate long-term safety of TNP and efficacy in decreasing pill burden	233 Screened, 20 excluded, 213 enrolled, 212 administered TNP, 154 completed the study $\bar{X\;}$PAS age (SD): 63.4 y (10.5), 61.3% male Inclusion criteria: aged ≥20 y, HD 3 times a week, dialysis vintage ≥ 12 wk, PB dose unchanged for 4 wk, Sp 3.5-7.0 mg/dL, unchanged vitamin D and calcimimetic dose for 4 wk, Kt/V ≥ 1.2 Exclusion criteria: PTH > 600 pg/mL, IBD, IBS with diarrhea, presence of diarrhea Discontinuation rate: 27.4%	TNP 5 mg twice a day + PB for 52 wk Final TNP dose after titration at wk 52: 5 mg: 24.0%, 10 mg: 19.5% 20 mg: 21.4% 30 mg: 33.1% Data assessed: % of patients who have ≥ 30% reduction in total pill number of PB and TNP from BL Δ in Sp at each time point compared with BL Safety (AE)	77.5% of participants decreased their pill burden by ≥30% at wk 52 (95% CI, 71.1-83.0; *P* < 0.0001) 51.9% of participants completely switched to TNP at wk 50 $\bar{X\;}$ Sp at wk 52 5.1 mg/dL, like BL, well-controlled even with a decrease in number of pills AE diarrhea: 63.7%, 74.8% mild	TNP is an effective way to reduce pill burden and control Sp in individuals with refractory hyperphosphatemia	Single-arm, open label means results were not validated against a PCO Study location limits generalizability to United States owing to cultural differences in diet Multiple PB—patients can switch between PB, adjusted by investigator, may have introduced bias
Nakayama et al,^22^ phase 3, open-label, single-arm, multicenter, Japan Funded by Kyowa Kirin Co Conducted between March and August 2022 Quality grade: +	To investigate safety and efficacy of TNP in Japanese PD patients prescribed PB	82 Consented, 28 excluded, 54 enrolled, 34 completed the study $\bar{X\;}$In mITT, age (SD): 65.1 y (10.5), 69.2% male Inclusion criteria: aged ≥20 y, receiving PD ≥ 12 wk, stable PB dose, Sp 3.5-7.0 mg/dL, increased sP to 6.1-10.0 mg/dL after start of washout and from pre-enrollment Exclusion criteria: HD or hemodiafiltration < 12 wk before screening, IBD, IBS with diarrhea, peritonitis, catheter-related infection ≤ 4 wk before screening, presence of diarrhea Discontinuation rate: 37%	TNP 5 mg twice a day for 16 wk Final TNP dose after titration at wk 16: TNP 30 mg: 30.8% TNP 20 mg: 19.2% TNP 10 mg: 26.9% TNP 5 mg: 21.2% No TNP: 1.9% Data assessed: $\bar{X\;}$ Δ in Sp at wk 8 compared with BL $\bar{X\;}$ Δ in Sp at each time point compared with BL Achievement of Sp in range of 3.5-6.0 mg/dL Safety (AE)	$\bar{X\;}$ Δ in Sp for wk 8 compared with BL was -1.18 mg/dL (95% CI, -1.54 to -0.81) $\bar{X\;}$ Δ in Sp for wk 16 compared with BL was -1.65 mg/dL (95% CI, -2.08 to -1.22) Achievement of Sp in range of 3.5-6.0 mg/dL at wk 8: 46.3% Achievement of Sp in range of 3.5-6.0 mg/dL at wk 16: 76.5% AE diarrhea: 74.1%	TNP is a safe and effective drug to manage hyperphosphatemia in Japanese PD patients	Single-arm study Small sample size Short study duration No randomization Study location limits generalizability to United States owing to cultural differences in diet
Gan et al,^19^ randomized, double-blind, phase 3 trial, multicenter, China Funded by Wanbang Biopharmaceuticals, Fosun Pharma Conducted between March 2021 and June 2022 Quality grade: +	To evaluate safety and efficacy of TNP in adults receiving HD with hyperphosphatemia	394 Screened, 243 excluded, 151 randomized, 147 completed the study TNP (n = 77) PCO (n = 74) ITT: TNP (n = 75) $\bar{X\;}$ age (SD): 53 y (10.9), 58.7% male, 100% Asian PCO (n = 72) $\bar{X\;}$ age (SD): 54 y (11.3), 62.5% male, 100% Asian Inclusion criteria: aged 18-80 y, HD 3 times a week, dialysis vintage ≥ 3 mo, ≥3 wk of stable dose of PB, Sp 4.0-7.0 mg/dL, ≥1.5 mg/dL increase in Sp after washout, Sp 9.0-10 mg/dL after 1-wk washout or 6.0-10 mg/dL after 2- to 3-wk washout, stable access, Kt/V ≥ 1.2, calcium > 8.4 mg/dL, stable vitamin D/calcimimetic Exclusion criteria: calcium < 8.0 mg/dL or > 11 mg/dL after washout, PTH > 1,200 pg/mL, metabolic acidosis, hypovolemia, IBD, IBS with diarrhea, planned kidney transplant/modality change, hepatic dysfunction, infection, life expectancy < 6 mo Discontinuation rate: TNP grp: 9% PCO grp: 15%	TNP 30 mg vs PCO twice a day for 4 wk Data assessed: difference in the Δ in $\bar{X\;}$ Sp at wk 4 compared with BL $\bar{X\;}$Δ in Sp at endpoint visit compared with BL in each group Achievement of Sp < 5.5 mg/dL Safety (AE)	TNP had greater LS $\bar{X\;}$ difference in reduction in Sp at the endpoint vs PCO (-1.17 mg/dL; 95% CI, -1.694 to -0.654, *P* < 0.001) Statistical significance in improvement in Sp in the subgroup analysis for adults ≥45 to <65 y with Sp BL < 7.5 mg/dL (no statistics provided) TNP, as compared with PCO, was more likely to achieve Sp < 5.5 mg/dL (44.6%-10.1%) AE diarrhea: TNP: 28.6% PCO: 2.7%	TNP is an effective way to reduce Sp in individuals receiving HD with hyperphosphatemia	Short study duration of 4 wk No information on final TNP titration dose States that only subgroup analysis for specified age grp significant for Sp reduction, but does not provide statistics Study location limits generalizability to United States owing to cultural differences in diet
Block et al,^18^ randomized, double-blind, placebo-controlled, phase 3 trial, multicenter, United States Funded by Ardelyx Conducted between January 2016 and January 2017 Quality grade: +	To test safety and efficacy of TNP in individuals receiving maintenance HD as monotherapy	673 Screened, 454 excluded, 219 randomized into RTP, 164 rerandomized into RWP, 152 completed both study periods RTP: TNP 3 mg twice a day (n = 74), TNP 10 mg twice a day (n = 73), TNP 30 mg twice a day (n = 72) RWP: PCO (n = 82), pooled TNP (n = 82) RTP: TNP 3 mg: $\bar{X}$ age (SD): 55.7 y (11.5), 62.2% male, 40.5% White, 54.1% African American, 17.6% Hispanic or Latino TNP 10 mg: $\bar{X}$ age (SD): 57.4 y (10.8), 46.6% male, 34.2% White, 61.6% African American, 11% Hispanic or Latino TNP 30 mg: $\bar{X}$ age (SD): 54.2 y (10.9), 67.6% male, 42.3% White, 56.3% African American, 25.4% Hispanic or Latino RWP: PCO $\bar{X}$ age (SD): 55.8 y (11.8), 53.7% male, 31.7% White, 62.2% African American, 14.6% Hispanic or Latino TNP $\bar{X}$ age (SD): 55.2 y (10.4), 63.4% male, 35.4% White, 62.2% African American, 19.5% Hispanic or Latino Inclusion criteria: aged 18-80 y, MHD ≥ 3 mo, PB Rx ≥ 3 times a day, Sp 4.0-7.0 mg/dL, stable vitamin D and calcimimetics ≥ 4 wk, after cessation of PB, increased Sp ≥ 1.5 mg/dL. RWP: completed RTP and ≥1.2 mg Sp reduction from BL to RTP end Exclusion criteria: PTH > 1,200 pg/mL, Sp > 10 mg/dL within 3 mo, bicarb < 18 mmol/L on 2 consecutive measurements, diarrhea, life expectancy < 6 mo Discontinuation rate: RTP: 25.1%, 3 mg: 23%, 10 mg: 26%, 30 mg: 26% RWP: 7.3%, PCO: 10%, TNP: 4%-6%	RTP: TNP 3 mg, 10 mg, 30 mg twice a day for 8 wk (was originally a phase 2 dose-range trial) RWP: rerandomized to receive the same TNP dose or PCO for 4 wk $\bar{X}$ TNP dose after titration: 24.4 mg twice a day for SAS and ITT $\bar{X}$ TNP dose after titration: 22.8 mg twice a day for secondary efficacy analysis set Data assessed: $\bar{X}$ Δ in Sp from BL compared with the end of 8-wk RTP $\bar{X}$ Δ in Sp from the end of RTP compared with the end of RWP in pooled TNP grp vs PCO grp Proportion of patients with Sp < 5.5 mg/dL at each visit during RTP Safety (AE)	RTP: significant Δ in Sp from BL to end of 8-wk RTP: 3 mg: -1.05 (95% CI, -1.38 to -0.72; *P* < 0.001) 10 mg: -1.07 (95% CI, -1.40 to -0.73; *P* < 0.001) 30 mg: -1.09 (95% CI, -1.43 to -0.74; *P* < 0.001) RWP: increase in Sp 0.85 ± 1.68 mg/dL in PCO grp vs 0.02 ± 1.63 mg/dL in TNP grp, LS $\bar{x}$ difference -0.72 mg/d/L (95% CI, -1.19 to -0.25; *P* = 0.003) RTP: proportion of patients with Sp < 5.5 mg/dL at each visit: 3 mg: 28.8%-37.7% 10 mg: 24.6%-41.1% 30 mg: 25.0%-40.7% AE diarrhea: ∼40% (RTP)	TNP is a safe and effective way to manage hyperphosphatemia	Protocol was modified to a phase 3 trial at the request of the FDA and primary outcomes were changed In RWP, only “responders” were included limiting generalizability
Block et al,^17^ randomized phase 3 trial, multicenter, United States Funded by Ardelyx Conducted between January 2018 and February 2020 Quality grade: +	To assess long-term safety and efficacy of TNP in treating hyperphosphatemia in dialysis patients, the PHREEDOM trial	1,559 Screened, 995 excluded, 564 randomized, 255 rerandomized, 205 completed the study, 109 completed the safety control RTP: TNP (n = 423) SEV (n = 141) RWP: TNP (n = 128) PCO (n = 127) SEV (n = 117) SEP: TNP (n = 222) SEV (n = 112) $\bar{X\;}$RTP: age (SD): 58 y (13), 64% male, 47% White, 46% African American, 8% other. 28% Hispanic or Latino RWP: $\bar{X\;}$ age (SD): 57 y (12), 62% male, 45% White, 49% African American, 6% other, 28% Hispanic or Latino $\bar{X\;}$SEP: age (SD): 58 y (12), 63% male, 45% White, 49% African American, 6% other, 27% Hispanic or Latino Inclusion criteria: aged ≥ 18 y, HD 3 times a week, dialysis vintage ≥ 3 mo (HD), ≥ 6 mo (PD), Sp 4.0-8.0 mg/dL at start, increased Sp ≥ 1.5 mg/dL post washout, Sp ≥ 6.0 mg/dlL and < 10 mg/dL Exclusion criteria: Sp > 10 mg/dL in past 3 mo, iPTH > 1,200 pg/mL, fluid overload, IBD, IBS with diarrhea RTP discontinuation rate: TNP: 39.5%, SEV: 17% RWP discontinuation rate: TNP: 22.7% SEV: 4.3% PCO: 22% SEP discontinuation rate: TNP: 7.7% SEV: 2.7%	RTP: TNP 30 mg twice a day for 26 wk or SEV (dosed per standard, 52-wk safety control) RWP: those that were in TNP grp for 26 wk, were rerandomized to receive TNP at same dose in RTP or PCO for 12 wk SEP: TNP 14-wk extension $\bar{X\;}$ TNP dose after titration: 24.4 mg twice a day 10 mg: 13% 20 mg: 30% 30 mg: 57% Data assessed: difference in the Δ of Sp at RWP end compared with BL in TNP vs PCO Δ in Sp at each visit compared with BL Safety (AE)	RTP In ITT (n = 407): TNP $\bar{X\;}$resulted in an Sp decrease of 1.4 mg/dL In ITT, the subset that had an Sp reduction of ≥1.2 mg/dL at end RTP end $\bar{X\;}$had an reduction of 2.5 mg/dL RWP In EAS (n = 131): LS $\bar{X\;}$ difference between TNP and PCO in Sp from BL to RWP end -1.4 mg/dL (*P* < 0.0001) In ITT (n = 243), LS $\bar{X\;}$ difference between TNP and PCO in Sp from BL at the end of RWP was -0.7 mg/dL (*P* = 0.002) AE diarrhea: 52% in RTP, 3% in RWP, 6% in SEP, 6% severe diarrhea	TNP is a safe and effective way to manage hyperphosphatemia in patients receiving dialysis	This study did not investigate add-on therapy, only monotherapy (SEV was only a safety control) Trial was largely nonblinded SEV was used as the standard of care, no other binder was allowed, unspecified dose
Pergola et al,^24^ randomized, double-blind, phase 3 trial, multicenter, United States Funded by “None,” trial sponsored by Ardelyx Recruited between February 2019 and July 2019 Quality grade: +	To determine safety and efficacy of adding TNP to stable PB doses in individuals receiving dialysis with hyperphosphatemia as dual therapy, AMPLIFY trial	511 Screened, 275 excluded, 236 randomized, 228 completed the study TNP + PB (n = 117) PCO + PB (n = 119) Overall $\bar{X}$ age (SD): 54.5 y (12.5), 59.1% male, 49.8% White, 43.0% African American, other 7.2%, 28.5% Hispanic or Latino Inclusion criteria: aged 18-80 y, HD dialysis vintage ≥ 3 mo or PD ≥ 6 mo, Sp 5.5-10 mg/dL, ≥1 PB, stable PB dose in previous 4 wk Exclusion criteria: PTH > 1,200 pg/mL, hypovolemia, history of IBS with diarrhea or IBD Discontinuation rate: 3.4% TNP + PB (n = 5) PCO + PB (n = 3)	TNP 30 mg + PB or PCO + PB twice a day for 4 wk TNP twice a day dose after titration: TNP + PB: 30 mg (55.6%) 20 mg (29.9%) 10 mg (14.5%) Data assessed: Δ in Sp from BL compared with wk 4 Achievement of Sp < 5.5 mg/dL at wk 4 Achievement of Sp < 5.5 mg/dL at each time point Safety (AE)	TNP + PB had a significantly higher LS $\bar{X}$ reduction in Sp from BL to wk 4 than the PCO + PB (-0.84 mg/dL vs -0.19 mg/dL; *P* < 0.001) TNP + PB had a higher % Sp of <5.5 mg/dL than PCO + PB Wk 1 (49.1% vs 21%; *P* < 0.001) Wk 2 (41.4% vs 23.5%; *P* = 0.003) Wk 3 (47.4% vs 17.6%; *P* < 0.001) Wk 4 (37.1% vs 21.8%; *P* = 0.01) AE diarrhea: TNP + PB: 40%	TNP is an effective add-on therapy in managing hyperphosphatemia	Short study period— perhaps some needed longer titration periods Multiple binders used
Silva et al,^26^ phase 3, open-label, multicenter, United States Funded by Ardelyx Study was registered on June 18, 2019 Quality grade: +	To evaluate efficacy and safety of TNP as a monotherapy or dual mechanism to achieve Sp within the reference range of 2.5-4.5 mg/dL, the NORMALIZE trial	172 Enrolled (already completed PHREEDOM), 124 completed NORMALIZE TNP/TNP + SEV (n = 111) SEV + TNP (n = 61) FAS: $\bar{X\;}$ age (SD): 56.8 y (12.9), 63% male, 44% White, 50% African American, 6% other, 30% Hispanic or Latino Inclusion criteria: participant in the PHREEDOM trial (see above for inclusion criteria) Exclusion criteria: see above, scheduled for kidney transplant, planned change in dialysis modality, life expectancy < 12 mo Discontinuation rate: 28% TNP/TNP + SEV: 20% SEV + TEN: 43%	TNP/TNP + SEV: those who received TNP in RTP of PHREEDOM trial continued TNP (added SEV if Sp > 4.5 mg/dL) for up to 18 mo SEV + TNP: those who received SEV in RTP of PHREEDOM trial added TNP gradually, and decreased SEV if Sp ≤ 4.5 mg/dL for up to 18 mo Data assessed: Δ in Sp from PHREEDOM BL to each visit and NORMALIZE endpoint Achievement of Sp in target (2.5-4.5 mg/dL) at each post-BL visit and end visit (%) Achievement of Sp ≤ 4.5 mg/dL or <5.5 mg/dL at each post-BL visit and end visit (%) Safety (AE)	$\bar{X\;}$ Sp reduction from PHREEDOM BL to NORMALIZE end was 2.0 mg/dL Achievement of Sp in target (2.5-4.5 mg/dL) at end visit was 33% compared with 21% at BL Achievement of Sp ≤ 4.5 mg/dL ranged from 35%-49% at post-BL visits compared with 22% at BL Achievement of Sp < 5.5 mg/dL ranged from 56%-69% at post-BL visits compared with 44% at BL AE diarrhea: 22%	TNP is effective as a stand-alone therapy and a dual mechanism to achieve target Sp	Open label without control arm No details on final titration dose of TNP No details on time frame when the study was conducted Amendment with updated guidance on dosing only affected those who entered the study after its creation
Sprague et al,^27^ randomized, open-label study, multicenter, United States Funded by Ardelyx Conducted between September 2020 and March 2023 Quality grade: +	To evaluate different TNP initiation methods, evaluate effect of TNP as a core therapy and a combination therapy to achieve Sp ≤ 5.5 mg/dL, the OPTIMIZE study	535 Screened, 202 excluded, 333 enrolled in part A, 132 enrolled in part B Straight Switch (n = 151) $\bar{X\;}$ age (SD): 52.4 y (11.1), 70.9% male, 42.4% White, 43.7% African American, 2% American Indian or Alaskan Native, 8.6% Asian, 1.3% Native Hawaiian or other Pacific Islander, 2% other, 27.8% Hispanic or Latino PB Reduction (n = 152) $\bar{X\;}$ age (SD): 53.2 y (12.1), 67.1% male, 40.8% White, 46.7% African American, 3.9% American Indian or Alaskan Native, 2% Asian, 2.6% Native Hawaiian or other Pacific Islander, 3.9% other, 25% Hispanic or Latino PB Naive (n = 30) $\bar{X\;}$ age (SD): 55.4 y (16.5), 60% male, 66.7% White, 30% African American, 3.3% American Indian or Alaskan Native, 0% Asian, 0% Native Hawaiian or other Pacific Isalnder, 0% other, 43.3% Hispanic or Latino Inclusion criteria: aged 18-80 y, HD 3 times a week, dialysis vintage ≥ 3 mo (HD), ≥ 6 mo (PD), taking PB ≥ 3 times a day with Sp BL > 5.5 mg/dL and ≤ 10 mg/dL or PB naive with Sp > 4.5 mg/dL and ≤ 10 mg/dL, Kt/V ≥ 1.2 Exclusion criteria: Sp > 10.0 mg/dL in past 3 mo, PTH > 1,200 pg/mL, hypovolemia, history of IBS with diarrhea or IBD, scheduled for kidney transplant Discontinuation rate (owing to AE): 11%	Part A: Straight Switch: discontinued PB and started TNP 30 mg twice a day PB Reduction: decreased PB ≥ 50% and added TNP 30 mg twice a day Binder Naive: TNP 30 mg wice a day for 10 wk Part B: elective, 16-wk SEP Data assessed: Δ in Sp at each visit and endpoint compared with BL Achievement of Sp ≤ 5.5 mg/dL Achievement of Sp ≤ 4.5 mg/dL Safety (AE) Medication burden	Reduction in Sp: Straight switch: 0.91 ± 1.7 mg/dL PB Reduction: 0.99 ± 1.8 mg/dL PB Naive: 0.87 ± 1.5 mg/dL During the first 6 wk of treatment, LS $\bar{X\;}$ Δ in Sp in PB Reduction > Straight Switch at each visit (range, *P* = 0.0001 to *P* = 0.025) Sp ≤ 5.5 mg/dL at part A endpoint: Straight Switch: 34.4% PB Reduction: 38.2% PB Naive: 63.3% Sp ≤ 4.5 mg/dL at part A endpoint: Straight Switch: 11.9% PB Reduction: 15.1% PB Naive: 43.3% AE diarrhea: 39.9%	Different initiation strategies for TNP may be used as an effective means to lower Sp regardless of PB use	Open label, no placebo control Small study sample for PB Naive grp No information on final TNP dose after titration

Abbreviations: AE, adverse event; BL, baseline; CI, confidence interval; DM, diabetes mellitus; EAS, efficacy analysis set; FAS, full analysis set; FDA, Food and Drug Administration; GI, gastrointestinal; grp(s), group(s); HD, hemodialysis; HTN, hypertension; IBD, inflammatory bowel disease; IBS, irritable bowel syndrome; iPTH, intact parathyroid hormone; ITT, intention-to-treat; Kt/V, measure of dialysis adequacy; LS, least squares; mITT, modified intention-to-treat; MHD, maintenance hemodialysis; PAS, primary analysis set; PB, phosphate binder; PCO, placebo; PD, peritoneal dialysis; PTH, parathyroid hormone; RTP, randomized treatment period; RWP, randomized withdrawal period; Rx, prescription; SAS, safety analysis set; SD, standard deviation, SEP, safety extension period; SEV, sevelamer; Sp, serum phosphate; TNP, tenapanor; $\bar{X\;}$, mean; Δ, change.

a Quality grades were assessed using the Academy of Nutrition and Dietetics Evidence Analysis Quality Criteria Checklist for primary research.^36^

The 6 studies conducted in Japan had at least 1 overlapping investigator.^16^^,^^20^, ^21^, ^22^, ^23^^,^^25^ In an early open-label single-arm study, Akizawa et al^16^ tested the effectiveness of tenapanor on managing serum phosphate and reducing phosphate binder pill burden by giving participants 30 mg of tenapanor twice daily, in addition to their phosphate binders for 26 weeks without a placebo comparison group. Then, in a randomized, double-blind, placebo-controlled trial, Shigematsu et al^25^ included a placebo group and evaluated tenapanor 30 mg twice daily as an add-on therapy to phosphate binders for 6 weeks. Inaba et al^20^ compared multiple tenapanor groups that varied in dosage (5 mg, 10 mg, 30 mg, and 30 mg with titration) with a placebo for 6 weeks to determine appropriate dosing recommendations for hyperphosphatemia management. Nitta et al^23^ investigated uptitration with a tenapanor starting dose of 5 mg twice daily compared with a placebo for 8 weeks. Koiwa et al^21^ questioned the long-term safety of tenapanor and its effectiveness in attenuating the pill burden in an open-label, single-arm, phase 3 trial providing hemodialysis patients with tenapanor 5 mg twice daily in addition to phosphate binders for 52 weeks. The study by Nakayama et al^22^ was the only study to exclusively evaluate tenapanor in peritoneal dialysis patients in an open-label, single-arm, phase 3 study and provided participants with tenapanor 5 mg twice daily for 16 weeks. The study by Gan et al^19^ had a short treatment period but a robust study design as a randomized, double-blind, phase 3 trial comparing tenapanor 30 mg twice daily with a placebo in managing hyperphosphatemia in adults receiving hemodialysis.

Similar to the studies from Japan, all the 5 US studies also had at least 1 overlapping investigator but differed in study groups, drug dosages, and comparison study arms.^17^^,^^18^^,^^24^^,^^26^^,^^27^ Studies described randomization and stratification processes, and all, except 1,^26^ described the titration process of tenapanor. After the first study in the United States,^18^ the remaining trials were named AMPLIFY,^24^ PHREEDOM,^17^ NORMALIZE,^26^ and OPTIMIZE.^27^ The earliest study in the United States conducted by Block et al^18^ consisted of the following 2 study periods: (1) a randomized treatment period (RTP) and (2) a randomized withdrawal period (RWP). During the RTP, participants received tenapanor 3 mg, 10 mg, or 30 mg twice daily for 8 weeks.^18^ Then, the participants were randomized again in the RWP to receive a placebo or the identical tenapanor dose from the RTP for an additional 4 weeks.^18^ In contrast, the AMPLIFY study investigated a starting dose of 30 mg twice daily to a phosphate binder regimen compared with a placebo.^24^

Unlike the previous studies, the PHREEDOM study comprised the following 3 time periods: (1) the RTP, (2) the RWP, and (3) the safety extension period.^17^ During the RTP, participants were randomized to receive either tenapanor 30 mg or sevelamer carbonate for 26 weeks.^17^ After 26 weeks, the tenapanor group transitioned to the RWP and was rerandomized to receive either tenapanor at the same dose or a placebo for an additional 12 weeks.^17^ Those who completed the PHREEDOM trial were then eligible to enroll in the 18-month NORMALIZE trial in which those who received tenapanor in the PHREEDOM RTP continued tenapanor. Those who received sevelamer in the PHREEDOM RTP added tenapanor.^26^

In the OPTIMIZE trial, participants were randomized to either discontinue their phosphate binder and start tenapanor 30 mg twice daily (Straight Switch) (n = 151) or decrease their phosphate binder regimen by at least 50% and add tenapanor 30 mg twice daily (Binder Reduction) (n = 152).^27^ In addition, a smaller group (n = 30) not on phosphate binders (Binder Naive) started tenapanor 30 mg twice daily.^27^ The tenapanor starting dose for this review ranged from 5 mg to 30 mg twice daily, and investigators titrated the dose in the presence of abnormal serum phosphate levels or safety issues.^16^, ^17^, ^18^, ^19^, ^20^, ^21^, ^22^, ^23^, ^24^, ^25^, ^26^, ^27^

### Serum Phosphate Change

Although Akizawa et al,^16^ Koiwa et al,^21^ and Nakayama et al^22^ did not have placebo groups, these studies showed improved serum phosphate levels with tenapanor use. Akizawa et al^16^ reported a mean change (± standard deviation) in serum phosphate at the endpoint from baseline of -0.40 ± 1.15 mg/dL. Koiwa et al^21^ found that the study participants’ mean serum phosphate levels at the endpoint (5.11 ± 1.17 mg/dL) differed from baseline (5.26 ± 1.00 mg/dL). Researchers noted the minimal change in serum phosphate but suggested that pill burden reduction is invaluable even without drastic decreases in serum phosphate levels.^21^ Neither Akizawa et al^16^ nor Koiwa et al^21^ reported statistical tests for pill burden reduction. Nakayama et al^22^ found a more modest mean change of -1.18 mg/dL (95% confidence interval [CI], -1.54 to -0.81) at week 8 and -1.65 mg/dL (95% CI, -2.08 to -1.22) at week 16 compared with baseline.

The placebo-controlled Japanese studies reported significant between-group differences in the mean change in serum phosphate at the endpoint from baseline.^20^^,^^23^^,^^25^ In comparison with a placebo group, Shigematsu et al^25^ found significant reductions in serum phosphate at the endpoint from baseline in the tenapanor group, with a between-group difference of -2.07 mg/dL (95% CI, -2.89 to -1.26, *P* < 0.001). Nitta et al^23^ also found a statistically significant difference between tenapanor and placebo in serum phosphate change at the endpoint compared with baseline (-1.76 mg/dL; 95% CI, -2.16 to -1.37, *P* < 0.0001). Inaba et al^20^ observed the same between-group trend with each tenapanor dosage group (5 mg, 10 mg, 30 mg, and 30 mg with titration) compared with a placebo (*P* < 0.001). Gan et al^19^ had analogous findings in China; however, they observed a lower between-group difference than the Japanese studies (-1.17 mg/dL; 95% CI, -1.69 to -0.65, *P* < 0.001). In summary, all of the studies outside the United States^16^^,^^19^^,^^20^^,^^22^^,^^23^^,^^25^ found that tenapanor was effective in managing hyperphosphatemia.

In the US studies, investigators also found a reduction in serum phosphate with tenapanor use. Block et al^18^ found significant decreases (*P* < 0.001) in mean serum phosphate levels in the RTP at week 8 from baseline in tenapanor 3-mg (-1.05 mg/dL; 95% CI, -1.38 to -0.72), 10-mg (-1.07 mg/dL; 95% CI, -1.40 to -0.73), and 30-mg (-1.09 mg/dL; 95% CI, -1.43 to -0.74) groups and a significant least squares mean difference in the RWP when comparing tenapanor and placebo groups (-0.72 mg/dL; 95% CI, -1.19 to -0.25 mg/dL, *P* = 0.003). Similarly, in the PHREEDOM RTP, the mean change (standard deviation) in serum phosphate at week 26 was -1.4 (±1.8) mg/dL, and there was a significant least squares mean difference during the RWP when comparing tenapanor and placebo in the efficacy analysis set (-1.4 mg/dL, *P* < 0.0001) and in the intention-to-treat set (-0.7 mg/dL, *P* = 0.002).^17^

The AMPLIFY trial also found that the tenapanor group had a higher least squares mean reduction in serum phosphate at week 4 from baseline, compared with the placebo group (-0.84 vs -0.19 mg/dL, *P* < 0.001).^24^ Similarly, the NORMALIZE trial reported that the mean difference in serum phosphate reduction at the endpoint from the PHREEDOM baseline was -2.0 mg/dL in the full analysis set.^26^ Finally, Sprague et al^27^ found that the participants in the OPTIMIZE study achieved a mean (standard error) serum phosphate reduction of 0.91 (0.14) mg/dL, 0.99 (0.15) mg/dL, and 0.87 (0.27) mg/dL at the end of the 10-week treatment period in the Straight Switch, Binder Reduction, and Binder Naive groups, respectively; however, no statistical tests were performed. To summarize, the studies in the United States found that various doses of tenapanor reduced serum phosphate levels. Table 2 displays the mean differences between tenapanor and placebo.

**Table 2:** Tenapanor and Placebo Serum Phosphate Mean Difference^17^, ^18^, ^19^^,^^23^, ^24^, ^25^

Study	Mean Difference of Serum Phosphate at the Endpoint From Baseline Between Tenapanor and Placebo (95% Confidence Interval)	*P* Value
Block et al^18^	-0.72 mg/dL (-1.19 to -0.25)	*P* = 0.003
Block et al^17^	-0.67 mg/dL (-1.07 to -0.24)	*P* = 0.002
Pergola et al^24^	-0.65 mg/dL (-1.01 to -0.29)	*P* < 0.001
Shigematsu et al^25^	-2.07 mg/dL (-2.89 to -1.26)	*P* < 0.001
Nitta et al^23^	-1.76 mg/dL (-2.16 to -1.37)	*P* < 0.0001
Gan et al^19^	-1.17 mg/dL (-1.69 to -0.65)	*P* < 0.001

### Achievement of Target Serum Phosphate

If tenapanor reduces serum phosphate, it may help achieve the target reference range outlined in the Kidney Disease Outcomes Quality Initiative (KDOQI) 2020 Update.^10^ Of the 12 studies reviewed, 9 assessed the efficacy of tenapanor in achieving serum phosphate goals.^18^, ^19^, ^20^^,^^22^, ^23^, ^24^, ^25^, ^26^, ^27^ Studies defined the target range differently. Benchmarks included serum phosphate ≤ 6.0 mg/dL,^20^^,^^25^ ≤ 5.5 mg/dL,^20^^,^^25^^,^^27^ < 5.5 mg/dL,^18^^,^^19^^,^^24^^,^^26^ 3.5-6.0 mg/dL,^22^^,^^23^ and ≤ 4.5 mg/dL.^26^^,^^27^

Shigematsu et al^25^ had a serum phosphate goal of ≤6.0 mg/dL and found that those who added tenapanor (n = 23) to their phosphate-lowering regimen were more likely to achieve target serum phosphate levels than those who added a placebo (n = 24) (87% vs 37.5%). Furthermore, when classified based on the dosage of tenapanor, Inaba et al^20^ observed that a higher dose led to more frequent achievement, as the percentage of participants meeting the goal in the placebo group (n = 41) was 12.2% compared with 40.5%, 43.9%, 66.7%, and 70.7% in the 5-mg (n = 42), 10-mg (n = 41), 30-mg (n = 42), and 30-mg (n = 41) titration groups, respectively.

Nitta et al^23^ and Nakayama et al^22^ used 3.5-6.0 mg/dL as their target reference range for serum phosphate. In the open-label, single-arm study by Nakayama et al,^22^ 46.3% of participants who received tenapanor achieved the target serum phosphate level at week 8, and the percentage increased to 76.5% at week 16. Further evidence provided by Nitta et al^23^ demonstrated that a higher percentage of participants in the tenapanor group (n = 81) achieved the target serum phosphate range compared with those who received the placebo (n = 83) (70.4% vs 28.9%).

Among the studies with a serum phosphate goal of ≤5.5 mg/dL, Shigematsu et al^25^ found that those who took tenapanor (n = 23) were more likely to achieve target levels than those in the placebo group (n = 24) (73.9% vs 25%). Although Inaba et al^20^ stated that efficacy points included meeting both ≤6.0 mg/dL and ≤5.5 mg/dL, they did not report any results related to the number of participants who achieved the goal of ≤5.5 mg/dL. Sprague et al^27^ found that the participants in the Binder Naive group (n = 30) were more likely to achieve target serum phosphate (63.3%) as compared with the Binder Reduction group (n = 152) (38.2%) and the Straight Switch group (n = 151) (34.4%).

Targeting a narrower serum phosphate goal of <5.5 mg/dL, Gan et al^19^ found that the tenapanor group (n = 75) had a higher percentage of participants meeting this goal compared with the placebo group (n = 72; 44.6% vs 10.1%), and Block et al^18^ found that the proportion of participants meeting the target goal for serum phosphate in the tenapanor groups was 28.8%-37.7% in the 3-mg (n = 74), 24.6%-41.1% in the 10-mg (n = 73), and 25.0%-40.7% in the 30-mg (n = 71) groups. Silva et al^26^ observed that in both combination groups of sevelamer and tenapanor, the serum phosphate goal was achieved in 56%-69% at study visits compared with 44% at baseline. Moreover, Pergola et al^24^ compared the percentage of participants who achieved a serum phosphate level within the target reference range and found significant differences between tenapanor and placebo groups each week.

Although Sprague et al^27^ tracked the serum phosphate achievement of ≤5.5 mg/dL, they also evaluated a more challenging goal of ≤4.5 mg/dL. Sprague et al^27^ found that those in the Binder Naive group (n = 30) were more likely to achieve serum phosphate in the target range (43.3%) as compared with the Binder Reduction group (n = 152) (15.1%) and the Straight Switch group (n = 151) (11.9%). Similarly, Silva et al^26^ found that the percentage of participants meeting the goal for serum phosphate at study visits ranged from 35%-49% compared with 22% at baseline. The findings of Sprague et al^27^ and Silva et al^26^ indicate that tenapanor can help reduce serum phosphate levels and promote the achievement of serum phosphate in the “normal” range of ≤4.5 mg/dL.

### Reducing Pill Burden

Pill burden is associated with limited adherence to phosphate binder prescriptions and may lead to poorer serum laboratory measurements.^37^^,^^38^ Akizawa et al^16^ chose a reduction of ≥30% in total phosphate-lowering pills as their desired outcome and found that at the end of the study, 72% (95% CI, 59.3-82.0, *P* < 0.001) achieved that goal compared with baseline. Furthermore, 52% (95% CI, 39.7-64.6, *P* < 0.001) achieved a reduction of ≥50%, and 28% (95% CI, 18.0-40.7, *P* < 0.001) completely switched from phosphate binders to tenapanor.^16^ Similarly, Koiwa et al^21^ found a significant reduction of ≥30% in the total number of pills in 77.5% of participants at the end of the study compared with baseline (95% CI, 71.1-83.0, *P* < 0.0001) and 51.9% of participants completely switched to tenapanor alone.

In the NORMALIZE study, investigators explored reductions in the sevelamer pill burden.^26^ The group that received sevelamer in the RTP of the PHREEDOM study and gradually added tenapanor had a mean decrease of 23% in daily sevelamer dose.^26^ Regarding pill burden reduction, 42% of these participants achieved ≥30% reduction in sevelamer pill burden, and 30% achieved ≥50% reduction in sevelamer pill burden by the end of the study.^26^ The OPTIMIZE participants in the Straight Switch group had a median decrease from 9 daily pills at baseline to 4 and a median percent reduction of 44.4% in the total number of phosphate-lowering medications.^27^ The Binder Reduction group had a median reduction (min-max) to 6 (2-20) daily pills from 9 (3-23) at baseline and a median reduction of 16.7% in the total number of phosphate-lowering medications.^27^

Overall, in the studies that investigated pill burden, the number of participants who achieved a reduction of ≥30% in the number of daily phosphate-lowering pills compared with baseline ranged from 42%-75%, and 30%-52% achieved a reduction of ≥50%.^16^^,^^21^^,^^26^^,^^27^ Furthermore, in the studies that evaluated switching to tenapanor, 28%-51.9% of participants prescribed phosphate binders converted to tenapanor alone.^16^^,^^21^

### Drug-Related Diarrhea

The prescribing information package insert states that diarrhea occurs in 43%-53% of individuals taking tenapanor, but the prevalence of severe diarrhea in data from 754 trial participants^17^^,^^18^^,^^24^ is <5%,^39^ as indicated by alterations in stool form or frequency or the Bristol Stool Chart.^40^ In this review, studies assessed diarrhea as a safety outcome.^16^, ^17^, ^18^, ^19^, ^20^, ^21^, ^22^, ^23^, ^24^, ^25^, ^26^, ^27^ In most studies, at least 40% of tenapanor recipients experienced diarrhea.^16^^,^^17^^,^^20^, ^21^, ^22^, ^23^, ^24^, ^25^^,^^27^ Of those, 6 studies observed diarrhea in at least 50%,^16^^,^^17^^,^^21^, ^22^, ^23^^,^^25^ 5 reported at least 60%,^16^^,^^21^, ^22^, ^23^^,^^25^ and 2 reported diarrhea in over 70% of tenapanor participants.^16^^,^^22^ This underscores the need for symptom management.

**Figure 2:**
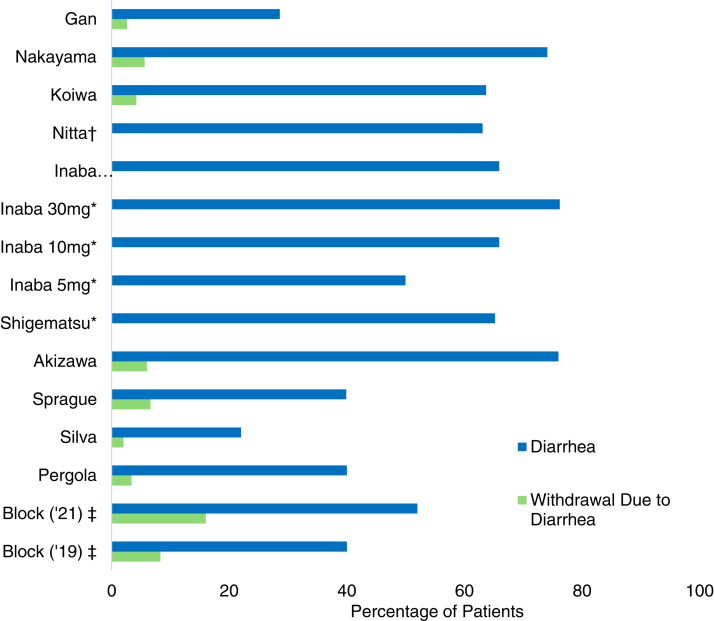
Diarrhea and tenapanor (references^16^, ^17^, ^18^, ^19^, ^20^, ^21^, ^22^, ^23^, ^24^, ^25^, ^26^, ^27^). ∗Inaba et al^20^ and Shigematsu et al^25^ did not disclose the exact percentages of participants who discontinued the study drug owing to diarrhea. ^†^Nitta et al^23^ reported that 0% of participants discontinued the study drug owing to diarrhea. ^‡^Random treatment period.

A concern with drug-induced diarrhea is the potential cessation of treatment, especially if the patient is unaware that the diarrhea is possibly transient.^17^^,^^24^^,^^26^ Block et al^18^ opined that the body may acclimate to the drug-induced changes in sodium and water content of stool. In the reviewed studies, 0%-16% of participants in tenapanor groups discontinued the drug because of diarrhea.^16^, ^17^, ^18^, ^19^, ^20^, ^21^, ^22^, ^23^, ^24^, ^25^, ^26^, ^27^ Fig 2 shows a comparison between the prevalence of diarrhea and the prevalence of diarrhea that resulted in drug discontinuation in all studies. The studies in this review had withdrawal rates between 3.4% and 35.8% owing to conditions including hyperphosphatemia, adverse events, personal reasons, kidney transplants, intolerable gastrointestinal side effects, and a breach at 1 study site.^16^, ^17^, ^18^, ^19^, ^20^, ^21^, ^22^, ^23^, ^24^, ^25^, ^26^, ^27^

## Discussion

According to the Dialysis Outcomes and Practice Patterns Study, 42% of hemodialysis and 46% of peritoneal dialysis patients have a serum phosphate level of >5.5 mg/dL.^1^ Given the association between hyperphosphatemia and morbidity and mortality,^41^ medication has played a central role in management, even in the absence of a randomized controlled trial demonstrating a link between phosphate-lowering medications and reduced morbidity and mortality. However, the pill burden of phosphate binders may contribute to hyperphosphatemia.^42^

This review substantiates that tenapanor is an effective drug for reducing pill burden, lowering serum phosphate, and fostering achievement of target reference phosphorus ranges in adults receiving dialysis with a grade II fair conclusion grade.^43^ The research explored tenapanor use as a monotherapy and adjunctive therapy, and evaluated ideal titration methods. Although these studies show promise in tenapanor use in managing hyperphosphatemia, tenapanor’s ability to mitigate fractures, cardiovascular events, and mortality is unknown.^18^^,^^44^

In addition, detrimental gastrointestine-related events were witnessed in all studies, prompting researchers to adjust dosages of tenapanor within study parameters. Final titrated doses, reported in nearly all studies, differed from initial starting doses and between participants based on tolerance and effectiveness. Investigators became aware of drug-related gastrointestinal consequences, prompting instructions to discontinue stool softeners before tenapanor use. Interestingly, Sprague et al^27^ provided participants with a questionnaire comparing hyperphosphatemia management before and after the study, focusing on bowel movements and pill burden, and 84% reported improvement compared with before the study.

Although the evidence from these studies suggests that tenapanor is effective for reducing serum phosphate, study limitations exist. Cultural practices and consumption of processed foods may differ between countries, deeming comparisons inappropriate. Furthermore, there were limitations regarding study design and methodology. First, a wide range of sample sizes and study durations impeded ascertaining a meaningful effect for long-term use in diverse populations. Second, although most inclusion criteria addressed the concurrent use of phosphate binders, not all participants were on the same phosphate binders within and between studies. Third, the trials did not track dietary intake, and hyperphosphatemia may indicate excessive dietary phosphate intake.^45^ Fourth, all the US studies^17^^,^^18^^,^^24^^,^^26^^,^^27^ had at least 1 overlapping author, as did the Japanese studies.^16^^,^^20^, ^21^, ^22^, ^23^^,^^25^ Although each study was appropriate to include, there is potential for bias. Finally, research funding for all studies was by either Ardelyx or their partners. Strengths of this review include homogeneous samples, clear protocol explanations, parallel methodologies, and clinically sound design methods.

### Practical Implications

The exhaustive effort to control serum phosphate in the dialysis population and the overwhelming medication burden make novel treatments appealing. If patients tolerate the accompanying drug-related diarrhea, then tenapanor can be an alternative option at the provider’s discretion. Tenapanor may exhibit compelling benefits in situations of refractory hyperphosphatemia and polypharmacy. In conclusion, this research supports the consideration of tenapanor in tandem with phosphate binders or as a monotherapy.

### Future Research

The prevalence of hyperphosphatemia in the CKD stage 5 population beckons the research community to explore the potential changes to tenapanor adherence over time. Longer studies, with more study groups receiving different dosages and permitting dose titration for diarrhea and serum phosphate control, may benefit people receiving dialysis. Additional explorations on the impact of inhibiting the sodium hydrogen exchanger isoform 3 on the absorption of other micronutrients are warranted. Newly designed studies by investigators that are not part of the drug approval process will limit bias. Finally, future studies should leverage the skills of the dietitian to provide nutrition counseling as a conjunctive therapy to advise on how to alleviate symptoms of tenapanor-induced diarrhea via diet and strategize on better serum phosphate management, both of which are likely to affect the roughly 555,000 individuals in the United States receiving dialysis.^46^
